# Case Report: Genetic testing reveals Wilson disease with familial hypertriglyceridemia in a 12-year-old boy

**DOI:** 10.3389/fped.2026.1763338

**Published:** 2026-03-06

**Authors:** Yuemiao Wang, Daren Wu, Dandan Sun, Jiawei Wang, Xun Wang

**Affiliations:** 1 Anhui University of Traditional Chinese Medicine, Hefei, China; 2 Department of Neurology, The Affiliated Hospital of Institute of Neurology, Anhui University of Chinese Medicine, Hefei, China

**Keywords:** copper metabolism, familial hypertriglyceridemia, genetic testing, lipid metabolism, wilson disease

## Abstract

Wilson disease (WD) and familial hypertriglyceridemia (FHTG) are both genetic metabolic diseases, and their comorbidity is extremely rare. This article reports a case of WD with FHTG in a 12-year-old Chinese boy. The patient was diagnosed due to elevated transaminase levels, combined with clinical manifestations, copper metabolism indexes, lipid profile analysis, and genetic testing results (pathogenic mutations of *ATP7B* and *APOA5*). The patient was treated using a copper chelating agent to lower copper levels and fibrate drugs to lower lipid levels, which resulted in improvements in his liver function and blood lipid indices. This case serves as a source of reference for the diagnosis and treatment of other similar cases. It not only reveals the potential interaction between copper metabolism disorders and lipid abnormalities, but also highlights the importance of systematic genetic testing to identify comorbid inheritance.

## 1 Introduction

Wilson disease (WD) is caused by a mutation of the *ATP7B* gene on chromosome 13, resulting in the inactivation of the ATP7B transporter protein, obstructing copper excretion in bile. Excess copper accumulates in the liver, brain, kidney, muscles, and other tissues, which can cause common liver diseases, cirrhosis, neuropsychiatric disorders (1). According to molecular genetic studies, the incidence of WD can be as high as 1/7,026 in some populations (2). Our department previously conducted a prevalence survey of WD in this region, which indicated an incidence rate of approximately 1 in 40,000 (3).

Familial hypertriglyceridemia (FHTG) is an autosomal dominant metabolic disease characterized by elevated triglycerides, with a prevalence of approximately 1 in 500 (4). The disease is caused mainly by gene mutations in the *APOA5*, *LPL*, and *GPIHBP1* genes, resulting in lipoprotein lipase system dysfunction, which significantly increases the risk of acute pancreatitis and atherosclerosis, and also leads to complications such as skin and eye lesions and hepatic steatosis (5).

Although WD and FHTG are both genetic metabolic diseases, their pathogenesis and clinical manifestations are different, and their coexistence is extremely rare (6). Liver damage caused by abnormal WD copper metabolism may disrupt lipid metabolism, whereas abnormal lipid metabolism caused by FHTG may also affect copper metabolism and excretion in individuals with WD. The combination of WD and FHTG makes clinical diagnosis and treatment challenging.

Therefore, a detailed history keeping, systematic laboratory tests, and comprehensive genetic testing are essential to confirm the diagnosis and develop an appropriate treatment plan. We report a rare case of a 12-year-old boy diagnosed with both WD and FHTG. We describe the diagnosis and treatment process to improve clinicians' understanding of how to approach genetic metabolic disease comorbidities.

## 2 Case description

In 2022, a 12-year-old boy was admitted to our hospital owing to persistent elevation of transaminase levels for 9 years. A review of his medical history revealed the following sequence of events:

In 2013 (at the age of 3 years), abnormal liver enzymes were detected as part of health screening by the school. His alanine aminotransferase (ALT) level was 162 U/L, and his aspartate aminotransferase (AST) level was 115 U/L; however, tests for hepatitis virus antibodies were negative. An abdominal color ultrasound revealed liver enlargement, and liver with increased echogenicity. After visiting a local hospital without a clear diagnosis and treatments such as “anti-inflammatory/transaminase-lowering treatment,” his liver enzymes did not improve. In July 2013, the patient was admitted to a local children's hospital. Investigations revealed elevated ALT (239 U/L) and AST (185 U/L) levels, a low ceruloplasmin (Cp) level (0.04 g/L; reference range: 0.2–0.6 g/L), 24-h baseline urinary copper excretion (off medication) was 123 µg. He was treated with oral penicillamine 0.375 g/d to promote copper excretion. Two months later, repeat liver enzymes tests revealed a decrease in his liver enzyme levels (ALT: 72 U/L, and AST: 63 U/L), and an increase in his 24-h urinary copper excretion to 407.4 μg. In October 2013, the patient attended our hospital for further copper chelation treatment, and tests revealed a persistently low Cp level of 0.04 g/L, and a 24-h urinary copper excretion of 387 μg. A corneal Kayser-Fleischer (K-F) ring test was negative and brain magnetic resonance (MR) revealed no abnormal signals in the basal ganglia area. And the Leipzig score was 4(Cp less than 0.1 g/L, 2 points; 24-h basal urinary copper excretion exceeding 100 µg, 2 points.) (7), after a diagnosis of WD was established, sodium dimercaptopropane sulfonate (DMPS) (0.125 g) was administered to promote copper excretion, and his liver enzymes improved after 4 courses of DMPS treatment.

From 2014 to 2017, the patient attended the hospital regularly for consolidation treatment. The 24-h urine copper level fluctuated between 280.89 and 531.58 μg, suggesting that the urinary copper excretion treatment continued to be effective. Between treatments, the patients adhered to regular medication and a low-copper diet. In 2017, the patient attended our hospital for a re-examination of his copper biochemistry. This revealed a serum copper level of 1.41 μmol/L, copper oxidase level of 0.029 U/L, and a C0p level of 0.046 g/L. He had an ALT level of 55 U/L, and an AST level of 48 U/L. The liver fibrosis indices were: hyaluronic acid (HA) 18.25 ng/mL, laminin (LN) 34.84 ng/mL, and type IV collagen (CIV) 29.68 ng/mL. Abdominal color ultrasound showed changes suggestive of diffuse liver disease (a starry sky appearance), but the spleen was stable in size. No palpable hepatomegaly or splenomegaly were present. Physical examination showed no abnormalities in muscle tone, tendon reflexes, and no corneal K-F rings. His overall condition remained stable with no new clinical manifestations.

In 2018 (at the age of 8 years), characteristic dyslipidemia was first detected during routine copper removal therapy. The liver enzymes indicators were: ALT 52 U/L, and AST 48 U/L. The blood lipid levels were: total cholesterol (TC) 161.99 mg/dL, triglyceride (TG) 549.5 mg/dL, high-density lipoprotein cholesterol (HDL-C) 30.15 mg/dL, and low-density lipoprotein cholesterol (LDL-C) 62.24 mg/dL. At the time, the abnormal lipid levels were attributed to the combined effect of abnormal copper metabolism secondary to WD and poor eating habits. No specific intervention was given to treat the dyslipidemia because the patient's liver enzymes were stable and he had no other risk factors for dyslipidemia. The clinician recommended adjusting the diet and monitoring the lipid levels regularly.

In 2019 (at the age of 9 years), the liver enzymes indicators were further improved: ALT level 52 U/L, AST level 42 U/L, but the dyslipidemia persisted, with a TC level of 166.24 mg/dL, TG level of 39.65 mg/dL, HDL-C level of 32.09 mg/dL, and LDL-C level of 67.27 mg/dL. From 2019 to 2022, the patient did not attend scheduled follow-up visits, but his parents reported that he had been adhering to oral copper chelation treatment without experiencing significant discomfort.

**Figure 1:**
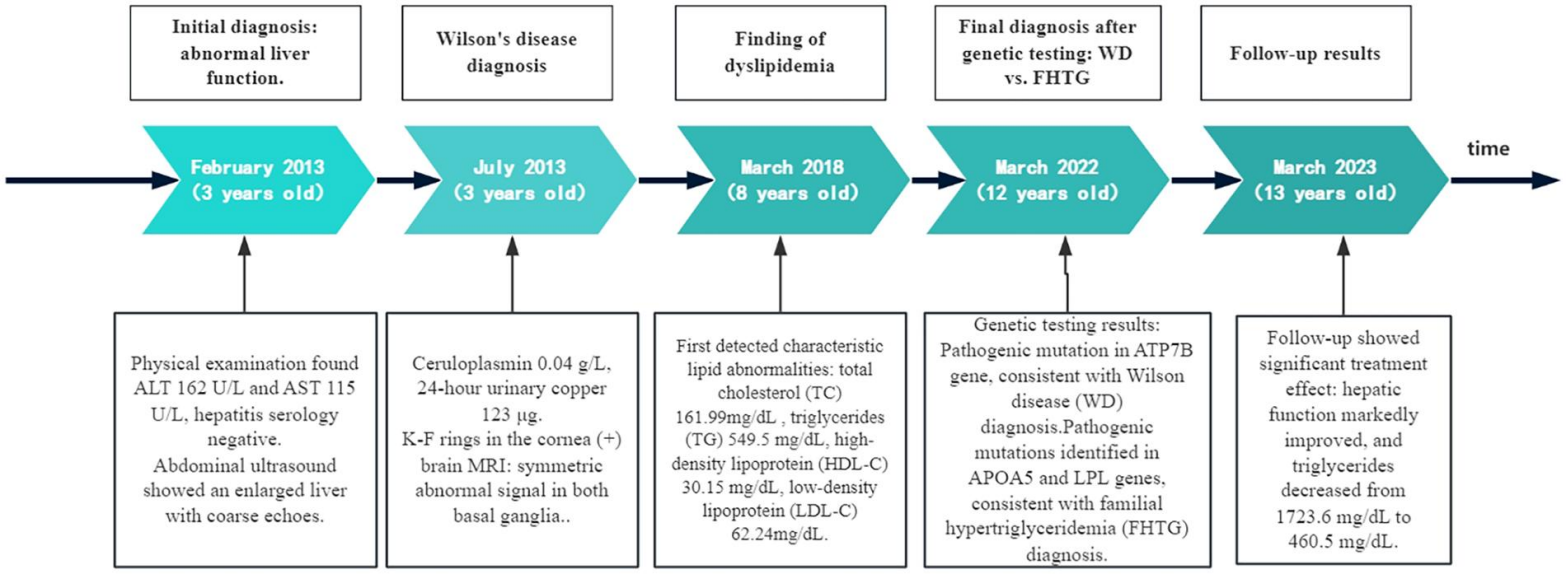
Timeline of clinical course and diagnosis: A case of Wilson disease with familial hypertriglyceridemia. ALT, alanine aminotransferase; AST, aspartate aminotransferase; FHTG, familial hypertriglyceridemia; HDL-C, high-density lipoprotein cholesterol; LDL-C, low-density lipoprotein cholesterol; TC, total cholesterol; TG, triglycerides; WD, Wilson disease.

In 2022 (at the age of 12 years), a comprehensive review showed stable liver enzymes with an ALT level of 42 U/L, and an AST level of 42 U/L. However, the TC level had increased to208.82 mg/dL, and the TG level was1723.6 mg/dL, with some visible turbidity in the specimen. The clinical timeline is summarized in Figure 1, and the serial changes in liver enzymes and lipid levels are summarized in Table 1.

**Table 1:** Laboratory examination results of the 12-year-old male patient admitted for suspected wilson's disease and familial hypertriglyceridemia.

Age	ALT	AST	Cp	TC	TG	HDL-C	LDL-C
(years)	(U/L)	(U/L)	(g/L)	(mg/dL)	(mg/dL)	(mg/dL)	(mg/dL)
3	239	185	0.04	—	—	—	—
7	55	48	0.046	—	—	—	—
8	52	48	0.042	161.99	550.86	30.16	62.26
9	52	42	0.04	166.28	397.89	32.09	67.29
12	42	42	0.04	208.82	1,723.6	—	—
12	45	43	—	—	460.5	—	—

ALT, alanine aminotransferase (0–50 U/L); AST, aspartate aminotransferase(0–50 U/L); Cp, ceruloplasmin (200–600 mg/L); TC, total cholesterol (<170 mg/dL); TG, triglyceride (<150 mg/dL); HDL-C, high-density lipoprotein cholesterol (>40 mg/dL); LDL-C, low-density lipoprotein cholesteroll (<100 mg/dL).

**Figure 2:**
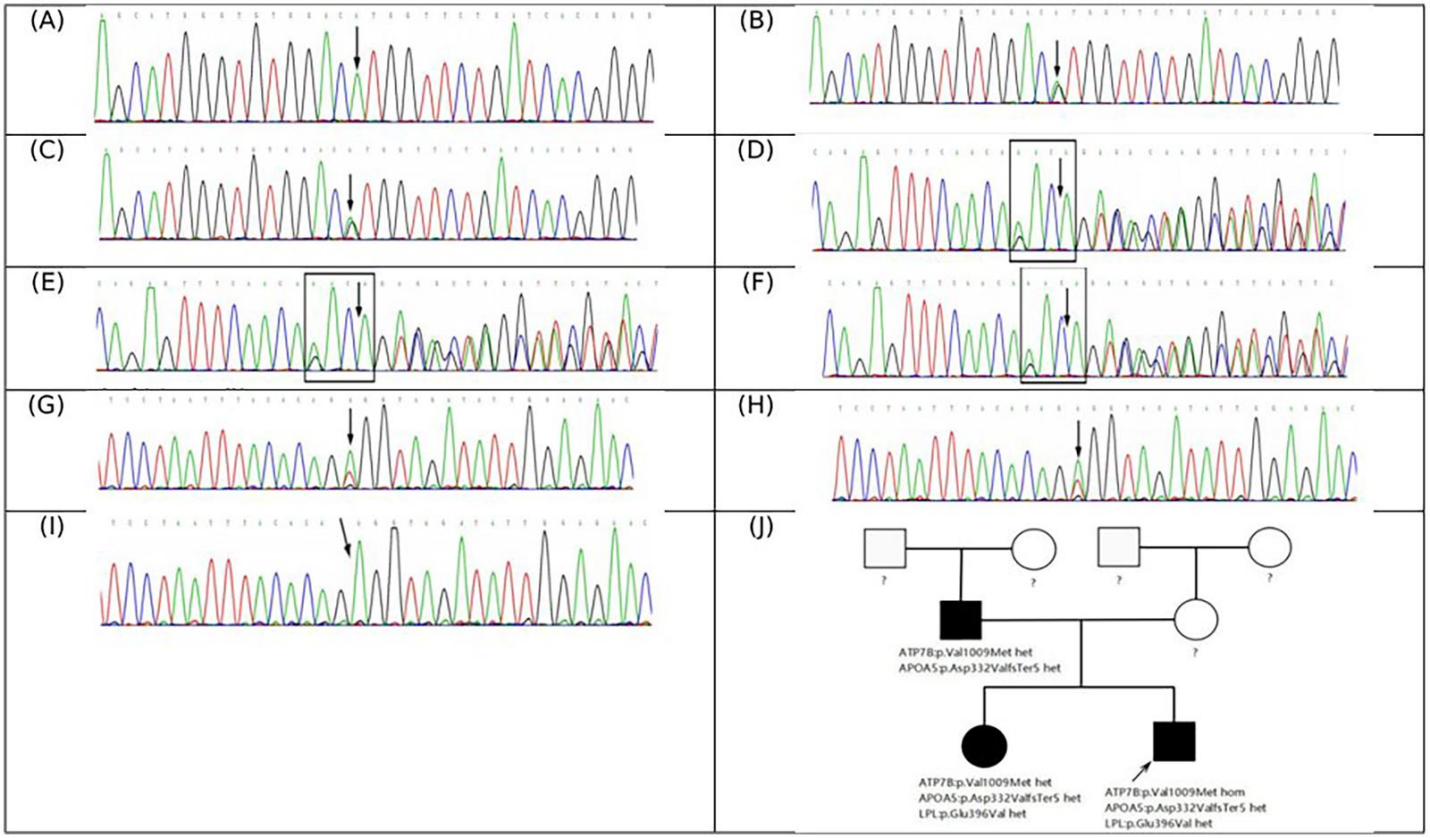
Mutation analysis of the *ATP7B* gene and related genes by next-generation sequencing (NGS), with validation by sanger sequencing. In the *ATP7B* gene, the proband **(A)**, his father **(B)**, and his sister **(C)** all carry the homozygous missense variant c.3025G>A (p.Val1009Met). In the *APOA5* gene, the proband **(D)**, his father **(E)**, and his sister **(F)**, all carry the heterozygous variant c.990_993del (p.Asp332ValfsTer5). Additionally, in the *LPL* gene, the proband **(G)** and his sister **(H)** both carry the heterozygous missense variant c.1187A>T (p.Glu396Val); however, the proband's father does not **(I)** Family pedigree **(J)** The question marks indicate unknown genotypes. The filled black symbols represent individuals with hypertriglyceridemia, and the black arrow identifies the proband.

In order to determine the cause of the dyslipidemia, a family investigation was conducted. This revealed that both the patient's father (aged 42 years) and sister (aged 14 years) had marked dyslipidemia. Genetic testing results showed that the patient had a homozygous mutation in the *ATP7B* gene c.3025G>A (Figure 2A), meeting the molecular diagnostic criteria for WD. Two additional pathogenic gene mutations were detected in the apolipoprotein A5 gene (*APOA5*) (c.990_993del; paternal source) (Figure 2D) and the lipoprotein lipase gene (*LPL*) (c.1187A>T; maternal source) (Figure 2G), which was consistent with the diagnosis of familial hypertriglyceridemia (FHTG). Pedigree verification showed that the father carried the *APOA5* mutation (Figure 2E) and the sister carried both the *APOA5* (Figure 2F) and *LPL* mutations (Figure 2H), and the genotype was consistent with the clinical phenotype.

## 3 Diagnostic assessment

The final diagnosis of this case was WD combined with FHTG. DMPS (0.25 g) was administered intravenously for 14 days to reduce copper levels in the body during hospitalization. Simultaneously, fenofibrate 160 mg/d was taken orally to lower lipids and the patient was managed with a strict low-copper and low-fat diet. After discharge, zinc gluconate (420 mg/d by mouth) was taken to inhibit intestinal copper absorption, and fenofibrate (160 mg/d by mouth) was continued to maintain lipid stability. The patient was followed up in March 2023 (age 12 years), and the laboratory test results showed a good response to treatment, with an ALT level of 45 U/L, AST level of 43 U/L, and TG level of 460.5 mg/dL.

## 4 Discussion

WD and FHTG are two independent genetic metabolic diseases, and although their simultaneous occurrence is rare, the combination of the two conditions has clinical significance and research value. WD is a copper metabolism disorder with various clinical manifestations of multisystem damage caused by abnormal copper accumulation. The clinical manifestations of WD are diverse and the initial manifestations in children differ from those in adults. In children, WD often first manifests as liver disease, and its clinical manifestations can range from liver enzyme abnormalities to cirrhosis and acute liver failure (8). In the nervous system, abnormal deposition of copper ions in the basal ganglia leads to extrapyramidal signs such as dysarthria, tremor, and dystonia, and some patients may experience mental symptoms such as anxiety and depression and WD can lead to intellectual disability (9).

FHTG is a common hereditary lipid metabolism disorder, and its core harm is to induce acute pancreatitis and accelerate the process of atherosclerosis. In addition, patients may also have characteristic clinical manifestations such as xanthoma, retinal lipemia, and hepatomegaly (5). The liver is the core organ of both copper and lipid metabolism (10, 11); therefore, when WD and FHTG occur together, each condition may exacerbate the other, and this increases the complexity of diagnosis and treatment.

Copper is related to lipid metabolism, and copper plays a two-way regulatory role in inhibiting synthesis and promoting decomposition by regulating copper-dependent enzyme activity under normal physiological conditions. Copper overload can inhibit hepatic lipid synthesis through multiple mechanisms (12), and this short-term metabolic effect may temporarily mask potential lipid metabolism abnormalities, as illustrated, at the time that the patient was diagnosed with WD copper accumulation continued, but only liver enzymes (ALT and AST) were abnormal, The patient's TG level was not elevated. In WD, early copy overload may temporarily mask the characteristic manifestations of FHTG. However, long-term high copper levels can lead to excessive accumulation of copper in hepatocytes, triggering oxidative stress responses that directly damage hepatocytes and further affect lipid metabolism (13, 14). Currently, there are no reports on the incidence of hepatic steatosis in Wilson's disease. Dyslipidemia or mild to moderate fattyliver is the first symptom to develop in many children withWilson's disease. Simple fatty liver patterns, steatohepatitis-like patterns, fibrosis, and cirrhosis can be observed in the liver pathology of adults/children with Wilson's disease. Furthermore, children with unexplainedfatty liver should be monitored for the possible development of Wilson's disease (15). Therefore, although high copper levels may initially inhibit lipid synthesis, the liver damage and oxidative stress caused by WD copper accumulation may further increase serum TG levels in patients with comorbid FHTG in the long term, increasing the risk of acute pancreatitis.

Lipid metabolism disorders in FHTG may also aggravate copper-metabolism disorders and liver damage due to WD, making WD condition more complex and severe. Lipid droplets formed by excessive TG can destroy the normal structure of hepatocytes and reduce the efficiency of copper bile excretion. Conversely, FHTG-related lipoprotein metabolism abnormalities may affect copper transport, and very low-density lipoprotein (VLDL) synthesis disorders not only lead to increased TG, but may also reduce copper-lipoprotein binding, reduce copper peripheral transport efficiency, and exacerbate hepatic copper accumulation (16).

Given the complexity of WD and FHTG comorbidities, regular monitoring of dynamic changes in blood lipids, improved apolipoprotein profiling, and comprehensive genetic testing are recommended for the diagnosis of such patients. In 2022, the patient completed relevant tests in our department, and the genetic test results showed that the child had a homozygous mutation of the *ATP7B* gene (c.3025G>A) combined with two additional pathogenic gene mutations on the *APOA5* gene (c.990_993del) and the *LPL* gene (c.1187A>T), which provided a key basis for the formulation of subsequent precision treatment plans. The results of family verification showed that both the father and sister of the patient had *APOA5* gene mutations and elevated triglyceride levels, demonstrating the value of family screening for the management of comorbidities.

In terms of treatment, balancing copper excretion and lipid-lowering is necessary to avoid placing an increased burden on the liver owing to the combined use of drugs. Although FHTG fibrates are effective in reducing triglyceride levels, they may increase the burden on the liver through hepatic metabolism (17). Therefore, we adopted a staged treatment strategy and gave priority to penicillamine, and sodium dimercaptosuccinic acid (DMSA) combined with zinc to control copper toxicity and stabilize WD. Once WD was stable, we gradually added fenofibrate in small doses to control the patient's TG levels. To optimize long-term disease management, we tested the patient's liver function and TG levels every 3 months and performed carotid ultrasound and liver elasticity testing every 6 months.

WD and FHTG comorbidities are relatively rare, and children with both conditions may have atypical clinical manifestations. Clinicians need to comprehensively and systematically evaluate the patient's history and conduct a thorough physical examination. Combined with a variety of molecular genetic tests, performing whole-exome sequencing as early as possible can help with early diagnosis and treatment and improve prognosis. Here, we report a case of a Chinese child with both WD and FHTG, highlighting the possible interaction between these two diseases. However, more clinical and basic research is needed to better understand the mechanisms by which they interact.

## Data Availability

The datasets presented in this study can be found in online repositories. The names of the repository/repositories and accession number(s) can be found in the article/Supplementary Material.
